# Editorial: Physiological and pathological changes of the retina associated with ageing

**DOI:** 10.3389/fncel.2025.1609473

**Published:** 2025-05-08

**Authors:** Ana I. Arroba, Eleni Beli, Jose R. Hombrebueno, María Llorián-Salvador

**Affiliations:** ^1^Institute for Biomedical Research and Innovation of Cádiz, University of Cádiz, Cádiz, Spain; ^2^Departamento de Endocrinología y Nutrición, Hospital Universitario Puerta del Mar, Cádiz, Spain; ^3^Wellcome-Wolfson Institute of Experimental Medicine, Queen's University Belfast, Belfast, United Kingdom; ^4^Department of Inflammation and Ageing, College of Medicine and Health, University of Birmingham, Birmingham, United Kingdom; ^5^Autonomous University of Barcelona, Barcelona, Spain; ^6^Vall d'Hebron Research Institute (VHIR), Barcelona, Spain

**Keywords:** retina, aging, age-related macula degeneration (AMD), glaucoma, senescence

Aging is a gradual, multifactorial process driven by time-dependent changes that progressively impair biological systems. In the nervous system, these alterations often lead to a decline in neuronal activity contributing to sensory deficits. Vision is particularly susceptible to age-related decline, which can severely impact on the quality of life by limiting daily activities, social engagement, and independence. The rapid expansion of the aging population poses major public health and socioeconomic challenges. By 2050, the proportion of individuals aged 65 and older is projected to increase from 9.3% in 2020 to 16%, significantly increasing the burden of vision-threatening conditions and associated healthcare cost (UN Population Division, 2020). Among these, age related macular degeneration, diabetic retinopathy, and glaucoma, leading causes of blindness worldwide, are expected to significantly contribute to this public health challenge (Teo et al., 2021; Wong et al., 2014; Tham et al., 2014). This Research Topic brings together four manuscripts that advance our understanding of how aging impact the healthy and disease retina.

The rod visual pathway undergoes significant structural remodeling with age. Changes are evident at the rod photoreceptor level, including shortening of outer segments, reduced opsin expression and axonal retraction. Second order neurons (including bipolar and horizontal cells), undergo compensatory sprouting, yet this remodeling appears insufficient to prevent age-related decline. In a longitudinal study of the aging mouse retina, Gierke et al. describe age-related changes in rod and cone photoreceptor ribbon synapses and postsynaptic neurons. Building on previous research (Sullivan et al., 2007; Terzibasi et al., 2009), they demonstrate synaptic plasticity in photoreceptors during aging through the formation of ectopic synapses between photoreceptors and second-order neurons. Interestingly, they report that synaptic remodeling during aging was not associated with changes in the protein composition of ribbon synapses, but rather with an increase in mitochondrial size in photoreceptor terminals. These findings are significant not only for understanding photoreceptor decline and synaptic remodeling during aging, but also for conditions that may accelerate retinal aging including diabetes (Hombrebueno et al., 2019; Crespo-Garcia et al., 2024), where disrupted mitochondrial homeostasis has emerged as a key factor in photoreceptor synaptic decline (Anderson et al., 2024).

Age-related macular degeneration (AMD) is one of the most prevalent visual conditions associated with aging (Wong et al., 2014). The pathogenesis of AMD is complex, with chronic inflammation playing a significant role, driven in part by microglial activation and cellular senescence, which exacerbate secretion of pro-inflammatory factors (Kauppinen et al., 2016). Among these, secreted phosphoprotein 1 (SPP1) has emerged as an important pathogenic mediator in inflammatory disorders (Wung et al., 2007; Chabas et al., 2001; Sato, 2005; Wong et al., 2005). To better understand the role of SPP1 in AMD, Lei et al. report a single-cell sequencing study of the human macula neuroretina. Their findings show a dominant upregulation of pro-inflammatory over anti-inflammatory cytokines in retinal microglia from AMD patients. Furthermore, they demonstrate that SPP1 is the most elevated senescence-related cytokines in both wet and dry AMD, which is associated with the pro-inflammatory and phagocytic status of microglia. This study underscores the pathogenic role of SPP1 in AMD and highlights its potential as a therapeutic target for this devastating visual condition.

While elevated intraocular pressure (IOP) is widely recognized as a major risk factor for glaucoma, aging independently contributes to ocular tissue vulnerability (Chang and Goldberg, 2012; Baudouin et al., 2021). People affected by glaucoma exhibit increased autoantibody titers against several proteins including heat shock proteins (HSPs) (Grotegut et al., 2020). HSPs maintain proteostasis by assisting in protein folding and degradation of misfolded proteins (Miyata et al., 2013). In glaucoma, elevated HSP27 expression and serum autoantibodies have been observed (Grotegut et al., 2020), while intravitreal HSP27 injection induces RGC cell loss independent of IOP (Grotegut et al., 2020). Building on these findings, Erb et al. sought to investigate whether age increases susceptibility to HSP27-induced glaucomatous damage. In their study, young (1–2 months) and older (7–8 months) mice received intravitreal injections of HSP27. No significant age-dependent differences were observed in the extent of RGC and optic nerve degeneration. However, older mice demonstrated a slightly heightened inflammatory response, as indicated by increased microglial activation. Further research on aged mice (16–18 month old) (Llorián-Salvador et al., 2024), is necessary to fully understand the role of HSP27 in age-related neurodegeneration and its potential contribution to glaucoma progression.

Extracellular matrix (ECM) is also affected by aging, leading to structural and biochemical alterations (Birch, 2018), which may compromise the homeostasis of retinal neurons. Accordingly, ECM alterations have emerged as important contributors of retinal disease, including AMD and diabetic retinopathy (Martins and Fernandes, 2023; Roy et al., 2016). Muller glia are critical for extracellular matrix remodeling (Limb et al., 2002), yet this function declines with age as shown by reduced production of ECM components and altered expression of matrix metalloproteinases. To further advance knowledge on this exciting research area, Prieto-López et al. present a comprehensive review of the role of Muller glia in shaping the ECM under physiological and pathological conditions. They also review the suitability of several biomaterials that mimic retinal ECM, positioning this review as a useful resource for refining *in vitro* platforms aimed at modelling ECM alterations in health and disease.

As the aging population and life-expectancy continues to grow, it is imperative to deepen our understanding of the molecular and cellular processes underpinning retinal aging and disease progression. Advancing this knowledge will offer new therapeutic avenues aimed at preserving vision, mitigate the healthspan-lifespan gap, and alleviate the societal challenges posed by age-related vision loss. We hope that this Research Topic has contributed meaningfully to clarifying key aspects of retinal aging, while highlighting the complex, multifactorial nature of age-related changes in both the healthy and diseased eye.
